# Anti-oxidant mediated normalisation of raised intracellular cytokines in patients with reproductive failure

**DOI:** 10.1186/s40738-018-0046-4

**Published:** 2018-03-02

**Authors:** Kevin Marron, John F. Kennedy, Conor Harrity

**Affiliations:** 1 Sims IVF Clinic, Clonskeagh Road, Clonskeagh, Dublin 14, Ireland; 2 Royal College of Surgeons Ireland, 123 St Stephen’s Green, Dublin 2, Ireland

**Keywords:** Immunomodulation, Cytokines, Implantation, Miscarriage

## Abstract

**Background:**

Raised intracellular cytokine ratios (CKR) are proposed as a significant risk factor for adverse reproductive outcome. An elevated cytokine ratio, such as between TNFa and/or IFNg to IL-10 is associated with recurrent miscarriage (RM). The use of pharmacological immunomodulators such as TNFα inhibitors in these patients is controversial and not generally recommended due to a lack of conclusive data supporting their use. We evaluated whether the use of anti-oxidants/dietary supplements as an alternative could positively influence CKR’s in ART patients.

**Methods:**

A prospective non-placebo control trial of antioxidant treatment for abnormal peripheral inflammatory cytokine ratios was performed. CKRs were assessed using flow cytometry in stimulated versus unstimulated whole blood samples in 337 IVF patients presenting with a previous history of poor outcome (RM or implantation failure). CKR’s were found to be elevated in 150/337. 70/150 patients in this elevated group agreed to a 10 week regime of Omega 3, vitamin D3, and B complex, followed by retesting to evaluate effect.

**Results:**

Mean cytokine ratios significantly improved between tests. Pre-treatment TNFa:IL-10 ratio improved from 71.6 to 21.0 (*p* < 0.0001) and IFNg:IL-10 ratio dropped from 24.5 to 12.5 (p < 0.0001). The improved ratios were achieved primarily by an increase in IL-10 expression (*P* = 0.0007), but also by a moderate decrease in stimulated TNFa expression (*p* = 0.008). Mean IFNg expression was unchanged (*p* = 0.42). On an individual basis CKR levels were normalised in 43 patients, improved in 12 and remained unchanged in 15. No significant differences in improvement were found between RM and IF subgroups.

**Conclusions:**

Intracellular cytokine expression levels and ratios were modifiable by the supplement regime employed. Elevated cytokine ratios have been linked with adverse reproductive outcomes, and proposed treatments have included biological immunomodulators which antagonise TNFa, but come with significant associated cost implications and more importantly, cytotoxic side-effects. A dietary regime is more patient friendly and lower risk, while still achieving a similar effect in many patients.

## Background

The interpretation and influence of the immune status of those with difficulties achieving or maintaining pregnancy, often with a background of prior unsuccessful assisted reproductive therapy, is controversial and not completely understood [1–4]. It is accepted that many factors such as embryo quality and genetic profile, timing of the transfer and a receptive endometrium are necessary for the successful initiation and maintenance of pregnancy [4]. Myriad other elements, including adhesion molecules, immune cells and various hormones must come together flawlessly, and often do. Confounders to this process are numerous, with advanced patient age and genetics, oocyte and sperm quality, tubal damage (hydrosalpinges), and thrombotic risk being noteworthy examples [5]. Circulating T lymphocytes, cytokine expression bias, and natural killer cell (NK) activity both within and outside the endometrium, are also all considered to be fundamental to successful implantation and maintenance of ongoing pregnancy [6, 7]. Increased expression of pro-inflammatory cytokines are thought to be the effectors in the immune compromised patient. It therefore follows that derangements, such as increased cytokine expression or an imbalance of pro-inflammatory versus anti-inflammatory elements, in these systems might also be partly responsible for immune cell mediated implantation failure or pregnancy loss. Some reproductive immunologists have believed these two negative outcomes are largely similar in certain patients, as both are potentially mediated by immune dysfunction or embryo aneuploidy, only separated by the timing of the event, with implantation failure occurring often before a pregnancy is biochemically detectable, and pregnancy loss a relatively common occurrence in up to 10% of all pregnancies in the first trimester [8, 9]. Cytokine ratios, calculated to represent the balance between pro- or anti-inflammatory components, can identify any bias, and have been regarded as a useful laboratory marker of derangements within the immune system in general [10–12]. By extension, it could be hypothesised that normalisation of these levels could potentially be associated with positive improvement in clinical outcomes for selected patients.

There is some evidence that in the assisted reproductive technologies (ART) setting the complementary use of appropriate anti-oxidants and dietary supplements may improve the spontaneous conception rate for sub-fertile couples, or may even help to increase the success rates of subsequent assisted reproductive treatments [13, 14]. A recent meta-analysis has shown that male anti-oxidant users are five times more likely to achieve a live birth with assisted reproductive technologies (ART) compared to placebo only, however, the effect of these therapies on female general subfertility, to date, is weak and inconclusive [15].

By focusing our observations on selected subpopulations with RM/IF, where potential immune derangement has been corroborated by baseline cytokine ratio elevation, specfic and appropriate patient subgroups have been identified for further study. Analysis of a specifically selected population, with hypothesised immune dysfunction, should hopefully bring some clarity to a possible role for Omega 3, Vitamin D and B complex to positively improve fertility, potentially by influencing intracellular cytokine ratios, the elevated presence of which has been associated with adverse fertility outcomes.

In the laboratory the ratios of the pro-inflammatory TNFa and IFNg to the anti-inflammatory IL-10 are given to represent the ratio between Th1 and Th2 cells, the balance of which is thought to be critical in both implantation and ongoing clinical pregnancy [6, 16]. In response to this hypothesis, TNFa antagonists such as Humira® (adalimumab) and Enbrel® (etanercept) have been controversially used in recurrent miscarriage or implantation failure patients [12] with varying degrees of success. These drugs should not be used lightly due to their side effect profile and potential toxicity, the extensive work up required to clear patients for their use, the lack of robust evidence to demonstrate benefit, and the absence of a pharmaceutical licence for their use in this field. Specific antioxidant and vitamin therapy could provide a novel and lower risk alternative to achieve these aims.

## Methods

Over a 3 year period (March 2013 to March 2016), patients with a history of repeated poor reproductive outcome were offered an initial assessment of their CD4+ intracellular cytokine ratios (CKR) if this was felt to be clinically appropriate (*n* = 337). Inclusion criteria for offering this test was a background of either miscarriage or implantation failure, with female age < 42 years, anatomically normal endometrial cavity on saline infusion sonogram, and normal endocrine profile (thyroid function and prolactin). Recurrent pregnancy loss (RPL) or recurrent miscarriage (RM) was defined as two or more failed clinical pregnancies [17]. The ASRM definition of clinical pregnancy (ultrasonic evidence) was applied [18]. Repeated implantation failure (RIF), is defined as > 2 unsuccessful blastocyst ETs along with a background history of primary infertility [19]. None of the treatment cycles incorporated pre-implantation genetic screening of embryos, as it was not performed in the Centre during the study time period.

**Fig. 1:**
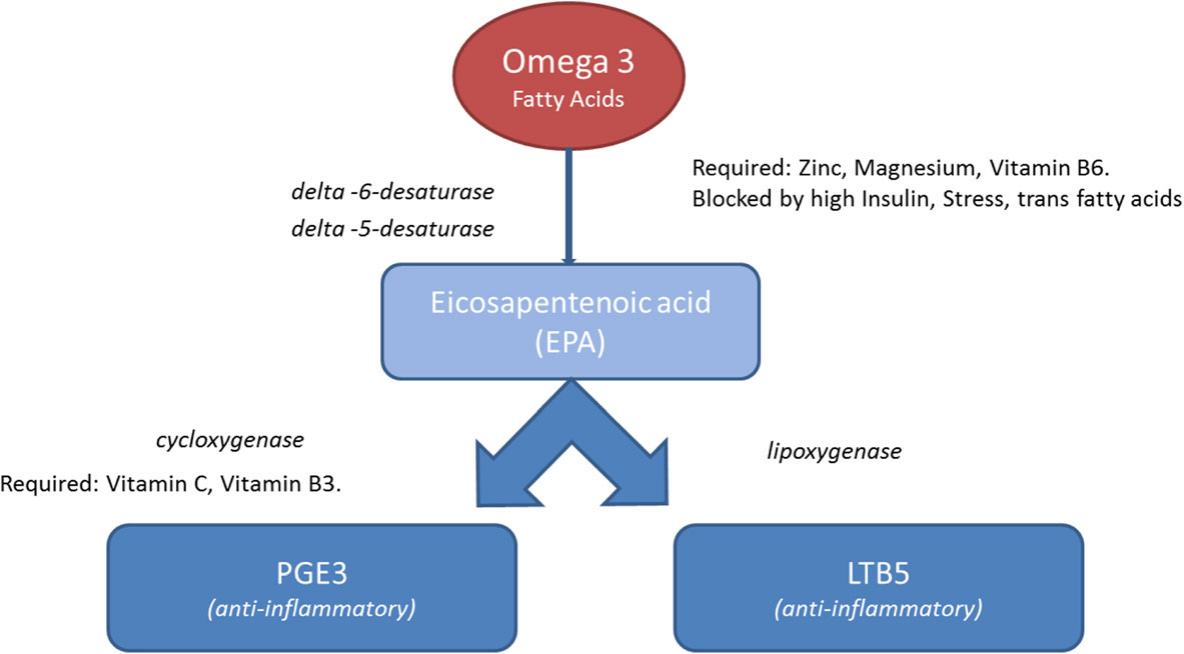
Potential biochemical pathways from omega 3 fatty acids to anti-inflammatory prostaglandins

**Fig. 2:**
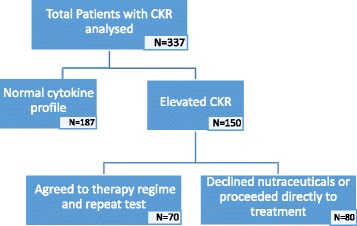
Patient enrolment breakdown

Normal ranges for assessing CKR’s were based on levels previously established by Rosalind Franklin Labs, Chicago, which are considered to be between 13.2 and 30.6 for IL-10:TNF-α and 5.8 to 20.5 for IL-10:IFN-γ [20]. In the laboratory the ratios of the pro-inflammatory TNFa and IFNg to the anti-inflammatory IL-10 are given to represent the ratio between Th1 and Th2 cells, the balance of which is thought to be critical in both implantation and ongoing clinical pregnancy [6, 16] These values are believed to be stable over several months in the absence of intervention. Patients with a stimulated intracellular cytokine ratio above these levels (*n* = 150) were offered a standardised 10 week regimen consisting of high dose omega 3 supplementation (3 mg/day), Vitamin D3 25 μg (1000iu) and B-complex with a view to beneficially modifying the biochemical pathways involved in inflammatory cytokine production (Fig. 1). This was followed by retesting of intracellular cytokine ratios after completion of the treatment protocol to determine if any effect was achieved. Seventy of the 150 patients (46.7%) agreed to treatment and re-testing. The remaining 80 patients either wished to proceed directly to ART without delay or declined nutraceutical adjuncts and/or repeat testing (Fig. 2).

### Cell separation and staining

**Fig. 3:**
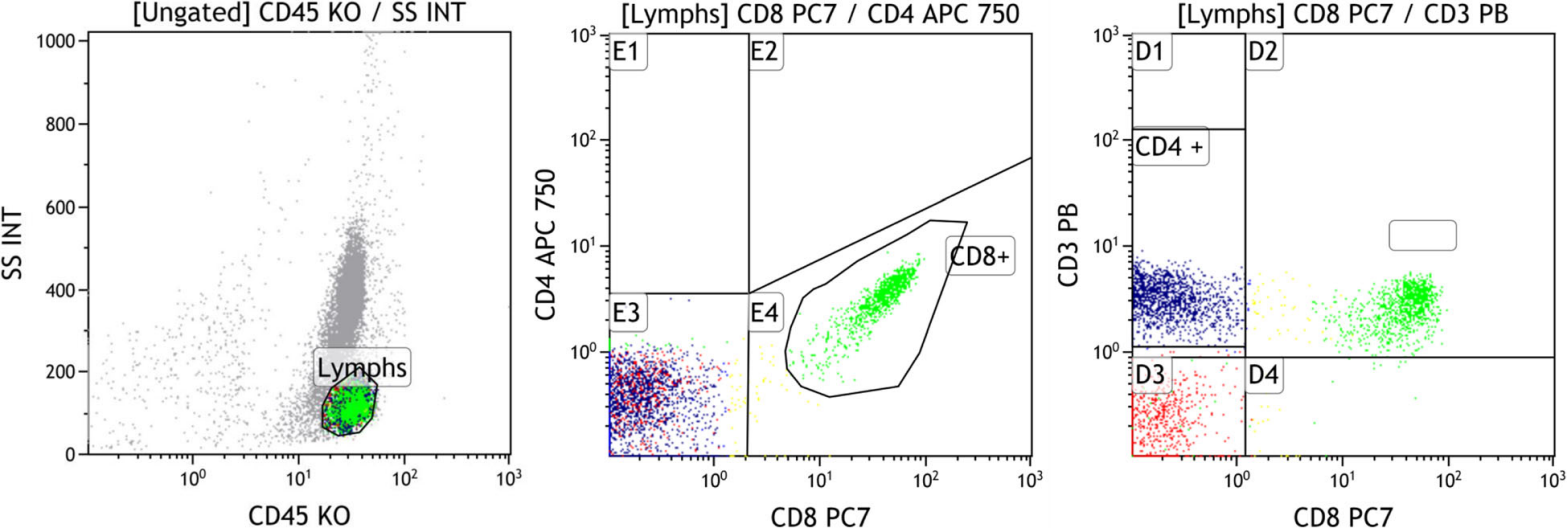
Gating strategy based on pan lymphocyte marker CD45+, with positive staining for markers CD3 and CD8 and negative gating to acquire the CD4+ T-lymphocytes. Intracellular cytokine expression is then measured in the CD4+ cells. This strategy is required as CD4+ surface marker expression is suppressed by the stimulatory process

**Fig. 4:**
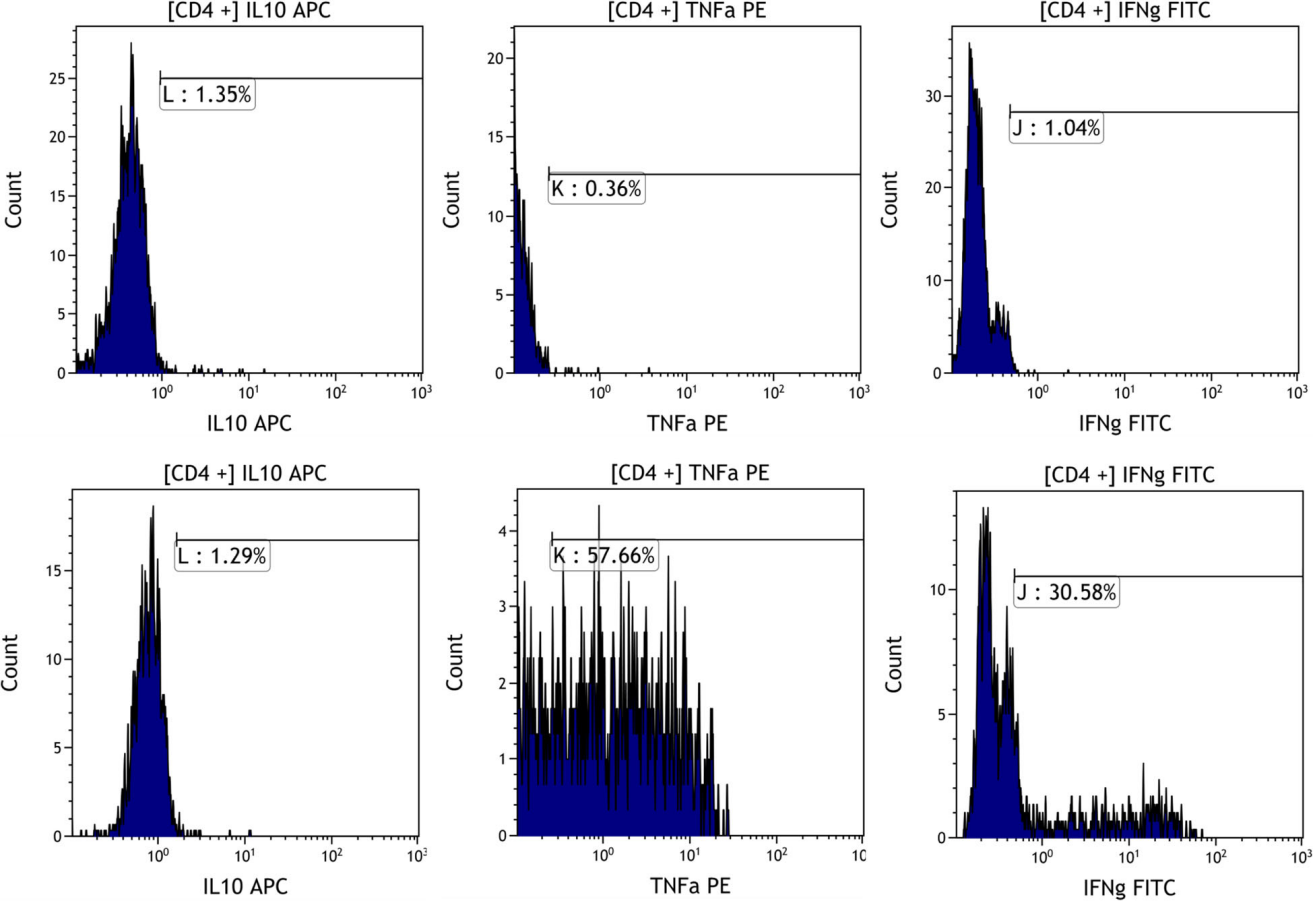
Top panel: Flow cytometry report showing unstimulated CD3+ CD8- (CD4+) T-lymphocytes. Correct gating on these cells allows for accurate positioning of the corresponding stimulated cell profile on middle panel. Thus the ratio of TNFa and IFNg expression to IL-10 expression can be calculated. In this case IL-10: TNFa is 1:43 and IL-10 to IFNg is 1:22. The lower panel indicates a typical response seen in a nutrient “responder” with an increase in IL-10 and a decrease in levels of response to TNFa and IFNg

Various in vitro methods have been reported for stimulating cytokine producing cells, this study mainly followed the protocol outlined by Kwak Kim [20] with the exception that whole blood was used in preference to ficol separated PBMC’s. Briefly, freshly collected whole blood cells were diluted into Iscoves modified Dulbecco’s medium (IMDM, Gibco Life technologies) fortified with 5000 IU penicillin & streptomycin (Gibco life technologies) and separated into stimulated and un-stimulated groups. The stimulated aliquot had 50 ng/ml phorbol myristate acetate (PMA) and 1 micromol/L ionomycin added in the presence of 1 uL Golgi Plug protein transport inhibitor to allow for protein accumulation in the Golgi complex. The unstimulated group had only the Golgi Plug introduced. Incubation took place overnight for ~ 18 h at 37° centigrade in 5% CO2. All cells were then processed and stained according to the manufacturer’s instruction with the BD whole blood Cytotoxic/Cytoperm kit (BD Pharmingen) Analysis on the Navios™ 10 colour 3 laser flow cytometer (Beckman Coulter UK) then followed immediately using dedicated software. Post incubation the cells were lysed in Pharmlyse buffer and following washing and centrifugation steps, were incubated with antibodies to surface ligands CD45 (anti-human Krome Orange, clone J.33), CD3 (anti-human Pacific Blue clone UCHT1) and CD8 (anti-human PC7 Clone B9.11), followed by a fixation and permeabilisation step. To detect intracellular cytokines, monoclonal antibodies to TNF-α, IFN-γ and IL-10 were employed, specifically phycoerythrin (PE)-anti-human TNF-α clone IPM2, Fluorescein isothiocynate (FITC)- anti- human IFN-γ clone 45.15 (both Beckman Coulter UK) and allophycocyanin (APC)-anti human IL-10 clone JES3-19F1 (BD Pharmingen). Corresponding isotype controls were utilized for each antibody and for each patient as well as fluorescence minus one (FMO) controls. It has previously been shown that following stimulation of lymphocytes with PMA and ionomycin, a down-regulation of the CD4+ moiety on the surface of lymphocytes occurs, therefore, a negative gating strategy was used to measure intracellular cytokine expression in CD3+ CD8- cells [20], hereafter called CD4+ cells. A region based on side scatter versus the pan WBC marker CD45 was used to demarcate the major lymphocyte population. This population was then used as the basis for all further evaluations (Fig. 3). We measured the percentage expression of our markers of interest in CD4+ T cells and from these values assessed the relative ratio of cytokine expression. CKR patients also act as their own experimental controls in that unstimulated blood is directly compared to stimulated blood, with the unstimulated levels giving the baseline values and levels above this baseline are attributed to the stimulation protocol (Fig. 4). IL-10 production is not affected by the stimulation protocol employed here.

Informed consent was obtained from patients for the analysis and approval was obtained from the Clinic’s research and quality management department. Statistical analysis, specifically paired Students t-tests were performed using SPSS v 21.0 to identify if any significant differences were noted pre- and post-therapy. This research did not receive any specific grant from funding agencies in the public, commercial, or not-for-profit sectors.

## Results

A total of 337 patients had a cytokine assessment over the study period, and 187 cases (55%) had no evidence of raised intracellular cytokine ratios, with levels either within (*n* = 147) or below (*n* = 37) the reported normal ranges. Cytokine expression (%) and calculated ratios for the total study population (*n* = 337) are described in Table 1. It was discovered that150 patients from the total cohort showed raised baseline intracellular cytokine ratios. Of these, 81 had raised TNFα:IL-10 ratios, with normal or low IFNγ:IL-10 (or “high/norm” classification) and 69 showing both raised TNFα:IL-10 and IFNγ:IL-10 ratios (“high/high” classification). From these 150 initial patients, 70 agreed to follow up with a second CKR evaluation approximately 10 weeks after the immunomodulatory regimen previously described (39/70 high/high, 31/70 high/norm) (Table 2). Further analysis of these patients revealed that 25 met the ASRM definition for recurrent miscarriage, and 33 meet the study design criteria to be categorised as implantation failure. It was found that 12 patients met the study inclusion criteria, but did not meet the strict definition for either of these 2 subgroups, and were classified as “other” (Table 2). The majority of patients had autologous own oocyte treatments/pregnancies, however, 8/70 had prior poor outcome using donor oocyte derived embryos. Post supplementation, considerable normalisation was achieved in the population, with 61% of patients (43/70) reaching either normal or normal/low levels for both markers (“norm/norm” classification). A further 12 (17%) patients were partially responsive to this particular treatment, with their mean cytokine ratios significantly improving between tests, but not reaching “norm/norm” status. Despite this therapy, 15 patients (21%) were unresponsive to the dietary regime with values remaining unchanged as either high/high or high/norm.

**Table 1:** Centile chart showing the initial CD4+ intracellular cytokine percentages and ratios for the entire dataset of 337 patients

		IL-10	TNFa	IGNg	TNFa/IL10	IFNg/IL10
		%	%	%	Ratio	Ratio
Centile	5	0.5	8.6	2.4	2.5	0.7
	10	0.5	13.0	3.6	3.6	1.3
	25	0.7	26.7	7.4	11.0	3.6
	50	1.3	43.4	13.9	26.7	8.6
	75	3.1	60.9	21.3	52.6	17.3
	90	8.1	72.6	31.4	78.3	32.8
	95	15.6	78.4	40.2	96.0	36.7
				n = 337

**Table 2:** The patient demographics of the observational group in whom intracellular ratios were outside the normal range on initial evaluation (n = 70) and who elected to have repeat intracellular cytokine measurements taken following a 10 week course of anti-oxidants and vitamins. The initial classification and obstetric history is also included

Patient demographics	
Overall Age (Mean ± SD)	37.3 ± 4.7
Overall Age (Median)	38
Classification	
Recurrent Miscarriage (RM)	25 pts
Implantation failure (IF)	33 pts
Other	12 pts.
Initial obstetric history RM group	
Age (Mean ± SD)	38.1 ± 4.9
Total pregnancies	85
Live births	9
Miscarriages	76
Miscarriage rate	89%
Initial obstetric history IF group	
Age (Mean ± SD)	35.9 ± 5.1
No. of embryo transfers	79
No. of pregnancies	0
Initial obstetric history “other” group	
Age (Mean ± SD)	39.3 ± 3.5
No. of embryo transfers	21
Total pregnancies	12
Live births	2
Miscarriages	10

**Fig. 5:**
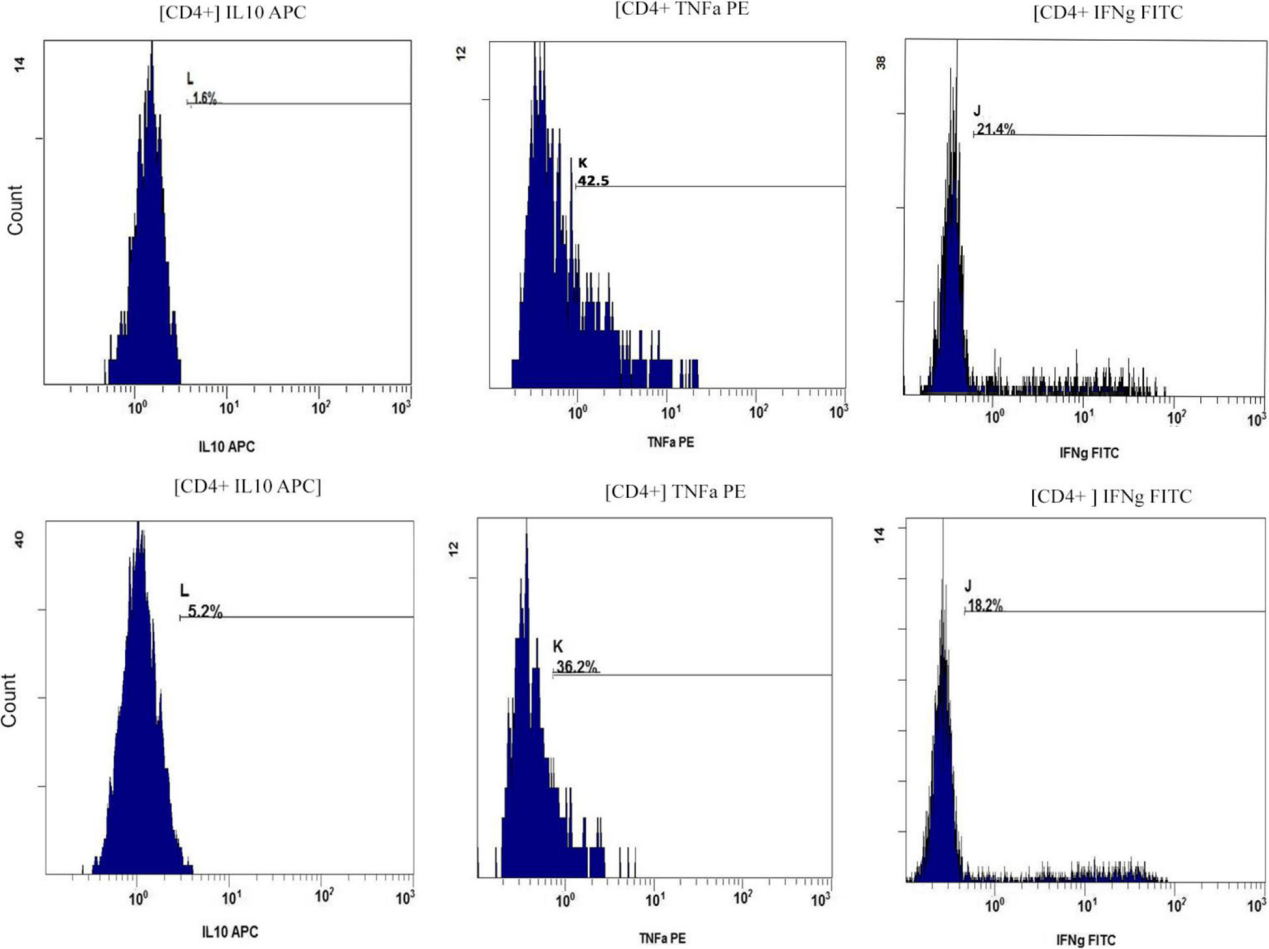
Top panel illustrates stimulated CKR levels prior to the 10 week nutrient regime. The lower panel illustrates the effect of the nutrient regime in the same patient approximately 10 weeks later. Percentage values in each case are relative to the paired unstimulated blood sample run concurrently to establish baseline expression

Pre-treatment mean TNFa:IL-10 ratio was particularly high in this overall population (*n* = 70) at 71.6, compared to the established normal range (13.2–30.6). Following the therapeutic regimen, mean ratios improved markedly to 21.0 (*p* < 0.0001). The initial mean IFNg:IL-10 ratio was also elevated in this group at 24.5 compared to the normal range (5.8–20.5). A similarly significant reduction to 12.5 (p < 0.0001) was observed post treatment. Improvements in both ratios with dietary nutraceutical treatment were primarily achieved by an approximately six fold increase in IL-10 expression. Initial levels changed from 0.8% (± 0.036 SEM) expression in CD4+ lymphocytes to 5.0% (± 1.18 SEM) post therapy (*P* = 0.0007). Interestingly, there was also a significant decline in absolute % expression for TNFα (*p* = 0.008), further improving the TNFa:IL10 with this treatment, however, stimulated expression of IFNg remained unchanged (*p* = 0.42) (Fig. 5) . A baseline pre-treatment comparison between RM and IF groups showed no discernible difference in expression of cytokine intracellular ratios between these two aetiologies, interferon gamma expression was marginally higher in the RM group (Table 3). For clarity, further pre- and post-nutraceutical analysis are presented for the RM/IF groups rather than the entire treated group (Table 4) Overall, normalisation of ratios was similar, and significant, in both groups. The changes in individual expression of TNFa and IFNg, however, were more marked in the implantation failure population, wih IFNg being potentially more resistant to change in those with miscarriage.

**Table 3:** Baseline cytokine percentage expression and ratios in the 70 person investigated group, illustrating little or no difference in stimulated intracellular cytokine expression between the two main populations; recurrent miscarriage and implantation failure

		RM	IF	*P value*
IL-10	%	0.9	0.8	0.331
TNFa	%	57.3	50.5	0.153
IFN-g	%	22.6	17	0.044
TNFa: IL10	Ratio	71	70.9	0.997
IFNg:IL-10	Ratio	26	23.2	0.332

**Table 4:**
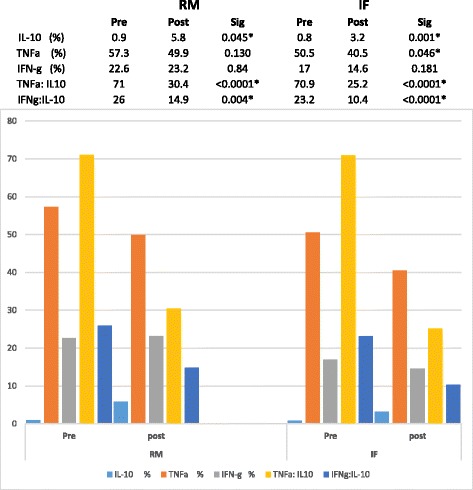
Table and graph illustrating average changes in the markers investigated, in both the RM and IF populations, pre- and post-micronutrient therapy. Asterix (*) denotes those values which were found to be statistically significant

## Discussion

Reproductive immunology is certainly on the fringe of accepted practice in the world of assisted reproduction, and for every advocate there are many naysayers [21]. The presence of a dominant Th1 type phenotype in patients with recurrent miscarriage and/or implantation failure, however, is often thought to be a common underlying pathology [20, 22, 23]. Controversy exists, however, as to the utility of immune testing in peripheral blood, and also its relevance to the uterine environment [2, 23–25]. Some of the techniques employed today to elucidate the immunological problems of selected IVF failures and recurrent miscarriers are, however, starting to gain traction. There are many methods available, looking at varied markers in both peripheral blood and endometrium, which assess either percentage expression or cell numbers [7, 26]. Specific measurement of intracellular cytokines, however, pre-and post-stimulation is quite controversial with many experts not advocating its use. Work in this field has linked elevated Th1:Th2 cytokine ratios with both recurrent spontaneous miscarriage and implantation failure [20]. It could be inferred that improvement in cytokine ratios may help improve reproductive outcomes in these patients, but there is a lack of evidence to investigate this hypothesis. A cited failing of many publications in this field, and perhaps the reason for the uncertainty associated with medical interventions in these patient populations, is believed to be poor initial patient selection. Given the multifactorial elements associated with RPL/IF the patient diversity is understandable. We believe the initial evaluation of intracellular cytokine levels and ratios is beneficial in identifying the population, in a largely heterogenous group, who may benefit from a specific therapeutic approach, namely more potent immune modulating therapies such as oral prednisolone and intralipid infusions. In our study, slightly less than half of the patients presenting with an RPL/IF phenotype met the criteria for inclusion, perhaps indicating the negative outcomes in the remainder were due to the vagaries of chance, genetics, anatomic, bacterial or viral issues.

A recent meta-analysis has shown that micronutrient therapy can improve outcomes in patients undergoing IVF [27]. There are many potential mechanisms of action for micronutrient therapy and the potential effect of these on cytokine ratios has not previously been explored. Omega 3 derived from fish oil, especially Eicosapentaenoic acid (EPA), has been shown to decrease NK cell activity by up to 48%. [28], and potentially also inhibit TNFa production, a potent pro-inflammatory cytokine, by as much as 74% [29]. A more recent study has linked Vitamin D deficiency directly with recurrent pregnancy loss [14]. Vitamin D is a well-established immune modulator and as such may have an influence on reproductive capacity. Indeed, deficiencies of vitamin D are connected to autoimmune problems in general [30]. Vitamin D has many and varied functions but in these cases it is thought it regulates the function of T helper cells and thus decreases T helper 1 (Th1) response, as well as promoting the suppressor T helper 2 (Th2) cells that help the body maintain a pregnancy [31]. Recent evidence points to female vitamin D deficiencies as predisposing patients to recurrent miscarriage by increasing the development of autoimmunity [14]. Murine studies have shown that making mice deficient in vitamin D makes them infertile [32]. The mediators of all these effects are thought to be modifications to the production and release of pro-inflammatory cytokines such as TNFa and IFNg, which are primarily the purview of CD4+ T cells and NK cells. In the analysis presented here, it has been shown that a broad spectrum nutraceutical regimen can be beneficial in significantly reducing IL10/TNFa ratios, as well as absolute TNFa expression by a possible immunomodulatory role. The potential effect of CKR normalisation on pregnancy outcomes with future ART has not been assessed here, but similar TNF a/IL-10 modifications alongside positive clinical pregnancy outcomes have been described in other populations [33]. Somewhat unexpectedly the significant improvement in cytokine ratios was not solely achieved by direct suppression of TNFa levels, but also by significant positive increases in the expression of the anti-inflammatory cytokine IL-10. This presents the intriguing possibility that this dietary regime may help to balance pro-inflammatory and anti-inflammatory cytokines in vivo. A potential benefit of nutraceutical regime employed is that it may achieve immunomodulation without perhaps compromising the ability to resist bacterial or viral infection, or leading to some of the numerous deleterious side effects associated with the use of the direct TNFa inhibitors Humira® and Enbrel® for example. Further research is needed into nutraceutical therapy for this purpose, but it is possible that the consideration of more toxic TNFa blockers could be limited to those who do not respond to a simpler intervention.

### Limitations of the study

This study is limited by a lack of a control group. Cytokine follow up on those patients with high initial intracellular cytokines who did not elect to have a repeat CKR evaluation post the proposed therapy regime, but instead elected to go straight to fertility treatment (*n* = 80), could have assisted with this. These patients, in general, went straight on to receive empirical treatment personalised to their history without delay. Any repeat analysis of the cytokines under these conditions would not have been informative for the purposes of studying dietary adjunctive therapy, as it would clarify that the normalisation was directly due to the regime and not random variance. To our knowledge there is no prior analysis on the consistency, or fluctuation, of stimulated cytokine expression or ratios over time. While not sufficient to constitute a control group, 7/80 patients who were not administered the supplementation regime as part of the study, were found to have had a future cytokine ratio retested, without any pre-analysis intervention or immunotherapy, at an average interval of 13 months. In this small subset of cases there was no significant difference in ratios at both time points, giving some support to the hypothesis that the effects demonstrated are due to the regime and not chance.

### Future areas of study

Endometrial tissue is increasingly regarded as the optimum tissue for investigation of immune cell dysregulation, unfortunately, in our hands the lymphocytes obtained from endometrial biopsies do not survive the aggressive stimulation process used in whole blood lymphocytes, indicating further work must be done to optimise the technique. One approach we are taking is to move away from the time and stimulation bound, CD4 specific, intracellular cytokines and move to the direct measurement of all resident cytokines irrespective of the cell of origin. We call this test the total cytokine load and it is currently being developed with very encouraging results.

## Conclusion

Elevated cytokine ratios have been linked with adverse reproductive outcomes. Proposed protocols have utilised biological immunomodulators, which antagonise TNFa. These agents have cost implications, and a potentially cytotoxic side effect profile, particularly with repeated or prolonged exposure. A regime with a lower risk profile could be potentially more patient friendly, however those who do not respond this regime may still require stronger treatments. We feel we have identified a population subgroup in which our approach is merited, and with appropriate and timely interventions can allow these patients to realise their strong desire to become parents.

## Abbreviations

APC: Allophycocyanin
ART: Assisted reproductive technologies
CKR: Intracellular cytokine ratios
EPA: Eicosapentaenoic acid
FITC: Fluorescein isothiocynate
IF: Implantation failure
IFNg: Interferon gamma
IL-10: Interleukin 10
IMDM: Iscoves modified Dulbecco’s medium
IVF: in-vitro fertilisation
NK cells: Natural killer cells
PE: phycoerythrin
PMA: phorbol myristate acetate
RM: Recurrent Miscarriage
RPL: Recurrent pregnancy loss
T cells: T lymphocytes
Th1: T helper 1
Th2: T helper 2
TNFa: Tissue necrosis factor alpha
